# Oncogenic signaling-mediated regulation of chromatin during tumorigenesis

**DOI:** 10.1007/s10555-023-10104-3

**Published:** 2023-05-06

**Authors:** Jahangir Alam, Md Nazmul Huda, Alan J Tackett, Sayem Miah

**Affiliations:** 1 Department of Biochemistry and Molecular Biology, University of Arkansas for Medical Sciences, Little Rock, AR USA; 2 Winthrop P. Rockefeller Cancer Institute, University of Arkansas for Medical Sciences, Little Rock, AR USA

**Keywords:** Signaling, Epigenetics, Carcinogenesis and metastasis

## Abstract

Signaling pathways play critical roles in executing and controlling important biological processes within cells. Cells/organisms trigger appropriate signal transduction pathways in order to turn on or off intracellular gene expression in response to environmental stimuli. An orchestrated regulation of different signaling pathways across different organs and tissues is the basis of many important biological functions. Presumably, any malfunctions or dysregulation of these signaling pathways contribute to the pathogenesis of disease, particularly cancer. In this review, we discuss how the dysregulation of signaling pathways (TGF-β signaling, Hippo signaling, Wnt signaling, Notch signaling, and PI3K-AKT signaling) modulates chromatin modifications to regulate the epigenome, thereby contributing to tumorigenesis and metastasis.

## Introduction

Cell signaling is the mechanism by which cells respond to environmental stimuli and initiate appropriate action by turning on or off different signaling pathways; thus, these pathways are crucial for regulating biological processes. Typically, the binding of ligands to membrane-bound receptors activates the signal transduction processes and triggers a cascade of signaling activities through numerous signaling molecules. Ultimately, signaling molecules coordinate with activators, coactivators, transcription factors, and chromatin remodelers to regulate downstream gene expression, and initiate appropriate physiological or cellular responses. These responses include development, cell proliferation, apoptosis, differentiation, cell cycle arrest, cell migration, epithelial–mesenchymal transition (EMT), cellular homeostasis, tissue repair, metabolism, and immunity [1]. It is now readily apparent that signal transduction pathways and their divergent effector-proteins are critical modulators of gene expression and are particularly important for various aspects of chromatin dynamics in gene regulation.

## Chromatin, histone modifications, and epigenetics

Chromatin is a higher-order, complex structure of DNA and histone proteins. A nucleosome—the fundamental unit of chromatin—is composed of approximately 145–147 base pairs of DNA wrapped around a histone octamer; this octamer is composed of 2 copies of each histone protein: H2A, H2B, H3, and H4. The nucleosomes are assembled into a compact structure—chromatin—that is further stabilized by the linker histone H1. This creates high-order structures known as chromosomes [2]. Presumably, access to the DNA sequence for transcription, DNA repair, and DNA replication is tightly controlled, and the accessibility of DNA sequence is mostly controlled by posttranslational modifications (PTMs) of histone proteins.

**Fig. 1:**
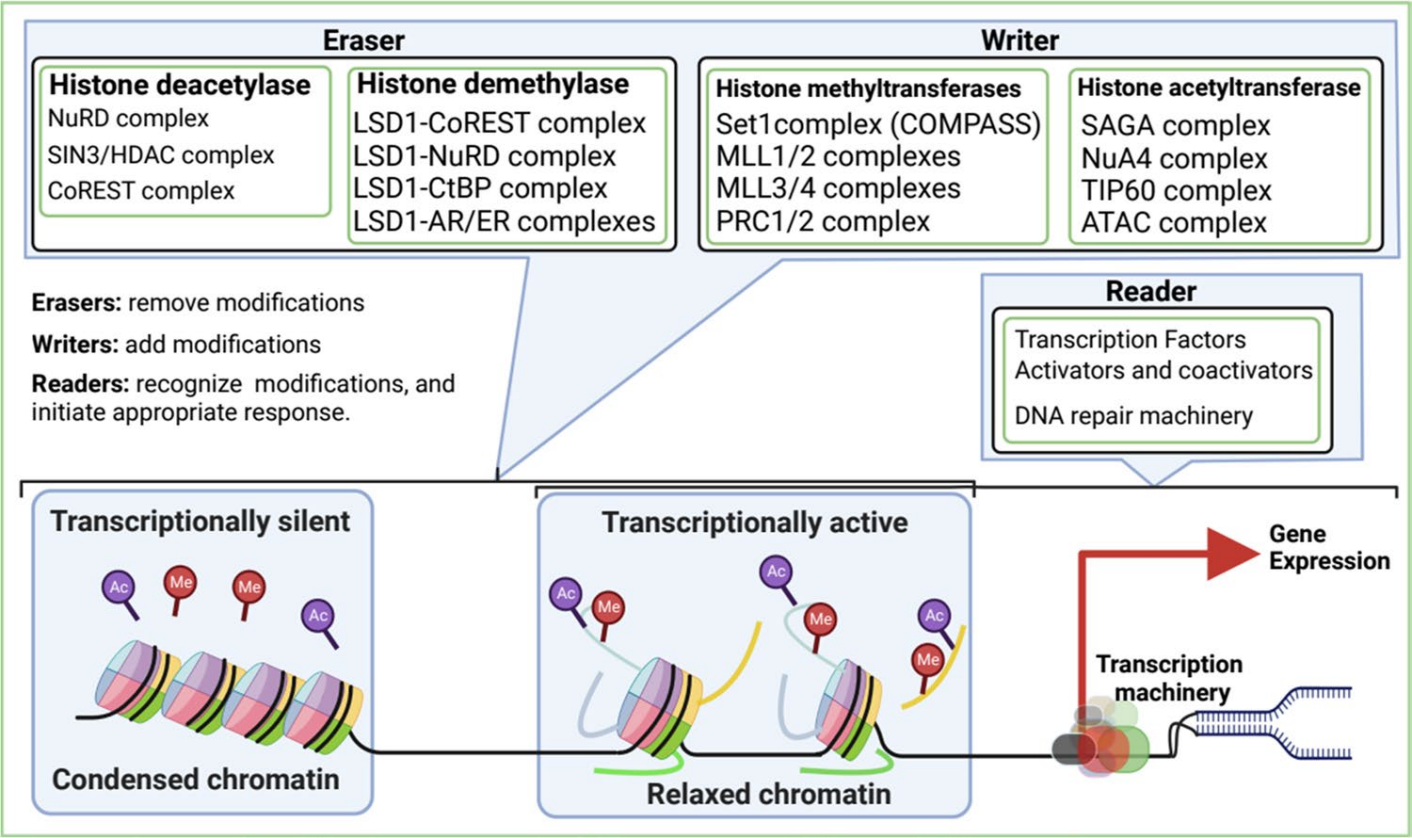
Model for histone modifications and functional consequences. Histone modifications alter the conformation of chromatin structure by either condensing the chromatin and inhibiting transcription (heterochromatin) or relaxing the chromatin and allowing transcription (euchromatin)

Although histones are mostly globular, the histone tails are unstructured and strikingly possess a large number and type of modifiable residues; these residues are the primary sites of PTMs (possible modifications and their biological functions are reviewed elsewhere [2, 3]). Histone proteins are dynamically modified by PTMs, and these modifications are associated with regulating chromatin structure and cellular functions. Different histone modification states (histone marks) have been implicated in the rearrangement of chromatin structure and recruitment of histone-related nonhistone proteins [3]. The group of histone-related proteins associated with histone modifications can be grouped into 3 primary classes: writers, readers, and erasers (Fig. 1). The histone-related proteins that add modifications to the histone tails are termed “writers,” the proteins that recognize specific modifications in histone tails are termed “readers,” and the proteins that remove modifications from the histone tails are termed “erasers.”

Chromatin can be in two distinct higher-order structures: the euchromatin state, which is an open and transcriptionally active structure, and the heterochromatin state, which is a condensed and transcriptionally inactive structure. PTMs on the histone tails regulate the euchromatin and heterochromatin states for different biological functions based on signaling stimuli [4]. For example, acetylation of histone’s lysine neutralizes the basic charge of the lysine, which loosens the interaction of the negatively charged DNA with histone; this allows an euchromatin (relaxed chromatin) structure. The euchromatin conformation increases the accessibility of histone-associated proteins, such as chromatin-remodeling complexes, transcription factors, cofactors, activators, coactivators, and other complexes for transcription. The fundamental role of chromatin-remodeling complexes in epigenetic and transcriptional regulation is important for many aspects of biological processes and diseases. In this review, we discuss different signal transduction pathways that dysregulate the canonical functions of chromatin-remodeling complexes to promote tumorigenesis and metastasis.

## TGF-β/SMAD signaling in chromatin regulation during carcinogenesis

The transforming growth factor-beta (TGF-β) superfamily of cytokines includes bone morphogenetic proteins (BMPs), growth and differentiation factors (GDFs), activins, inhibins, nodal, anti-Müllerian hormone (AMH), and TGF-β isoforms [5]. TGF-β is a pleiotropic, multifunctional cytokine (ligand) that is secreted by many cell types and plays a pivotal role in diverse biological processes, including cell differentiation and growth, migration, apoptosis, tissue homeostasis and repair, immune and inflammatory responses, and other cellular functions [6–8]. Mature TGF-β ligand binds with transmembrane serine/threonine receptor kinase TGF-β receptor II (TGF-βRII), which elicits phosphorylation of TGF-β receptor I (TGF-βRI) and subsequently forms a stable, active heteromeric complex of TGF-βRII and TGF-βRI. The activated TGF-βRII/TGF-βRI complex directly phosphorylates and activates R-SMAD proteins. SMADs are downstream effectors of TGF-β/SMADs signaling and can be grouped into 3 classes: receptor SMADs (R-SMADs: SMAD1, 2, 3, 5, and 8), inhibitory SMADs (I-SMADs: SMAD6 and 7), and the common mediator SMAD4 (co-SMAD). Upon phosphorylation and activation, the R-SMADs (SMAD2 and SMAD3) dissociate from the TGF-β receptor complex and subsequently form a complex with the common mediator SMAD4 [9], although SMAD4 is not obligatory for TGF-β signaling [10]. The SMAD2-SMAD3-SMAD4 complex then translocates into the nucleus and regulates gene expression, both positively and negatively [9]. The elegant simplicity of this core signal transduction pathway sharply contrasts with the intricacy of the elicited biological response.

The TGF-β/SMADs signaling transduction pathway has a pleiotropic role in cellular homeostasis and disease. It acts as a powerful tumor suppressor [11] in normal cells; however, in cancer cells, it promotes tumor progression and metastasis. How TGF-β/SMAD signaling becomes a tumor-promoting factor, given its antitumorigenic and antimetastatic functions in healthy cells, is a long-standing paradox in cancer biology. This signaling induces transcription of cell-cycle regulatory genes (i.e., P21, P16, PTK5) and downregulate pro-proliferative genes in healthy tissues; however, in cancer cells, it promotes EMT, invasion, and metastasis. Furthermore, tumor cells exploit the TGF-β/SMAD signaling to induce the angiogenesis program for tumor vascularization [12].

Failure or dysregulation of TGF-β signaling is involved in the development of several diseases. Dysregulation of TGF-β/SMAD signaling potentiates progressive renal injury and inflammation, and subsequently leads to chronic kidney disease [13]. Aberrant activity of TGF-β/SMAD2/3 signaling induces K17 overexpression, which contributes to the pathogenesis of psoriasis [14]. Dysregulation of TGF-β/SMAD4 signaling in smooth muscle cells triggers aortic wall inflammation, which leads to the pathogenesis of aortic aneurysms [15] and impairment of SMAD3 potentiates microglia-mediated neurodegeneration [16]. Several non-receptor protein tyrosine kinases have been associated with the regulation of TGF-β/SMAD signal transduction pathways such as SRC phosphorylates TGF-β type II receptor, which significantly enhances the TGF-β-induced EMT of mammary epithelial cells [17]. PEAK1 modulates canonical TGF-β/SMAD signaling to potentiate TGF-β–induced cell proliferation and EMT [18]. Recently, we also discovered that protein tyrosine kinase 6 (PTK6) (also known as breast tumor kinase, BRK) interacts with SMAD2/3/4 and phosphorylates SMAD4, which alters TGF-β/SMAD signaling and increases the metastatic potential of breast cancer cells [19].

**Fig. 2:**
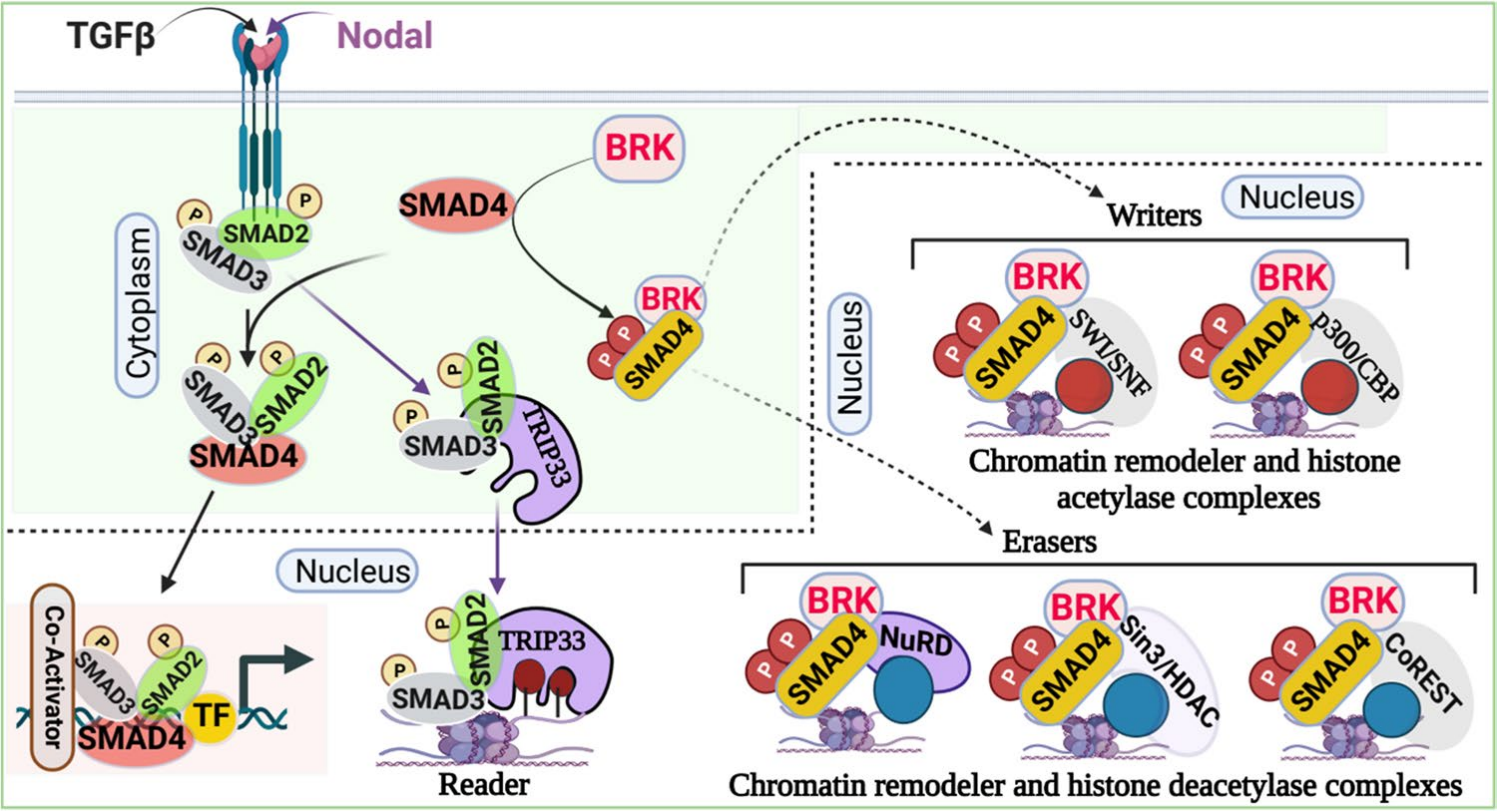
Canonical TGF-β/SMAD signaling and chromatin regulation. The downstream effectors of TGF-β/SMAD signaling—SMAD2, SMAD3, and SMAD4—take part in chromatin regulation and gene expression by interacting with chromatin-remodeler complexes and associated proteins. The dotted lines indicate further experimental validation is required

Even though TGF-β/SMAD signaling is inherently simple, the physiological functions of TGF-β/SMAD signaling are diverse and vary among different cell types and environmental conditions [20]. Combinatorial interactions in the heteromeric or heterotrimeric SMAD complexes, SMAD-interacting proteins, promiscuous protein–protein interactions with transcription factors, chromatin remodelers, and histone-modifying complexes allow TGF-β/SMAD signaling to become versatile and to diversify its biological functions [20, 21]. TGF-β signaling maintains cellular homeostasis through numerous mechanisms, such as inducing apoptosis, cell cycle arrest, EMT, and others [22, 23]. We recently discovered that PTK6 catalyzes the phosphorylation of SMAD4 at 2 tyrosine sites: Y353 and Y412 [19]; this induces interaction between phosphorylated-SMAD4 and the nucleosome remodeling and histone deacetylase complexes (i.e., Sin3/HDAC, NuRD, CoREST, SWI/SNF), suggesting a potential role in epigenetic reprogramming to regulate gene expression (Fig. 2).

NuRD, one of the major chromatin-remodeling complexes, is an important epigenetic regulator of gene expression in mammalian cells [24]. The NuRD complex regulates transcription through chromatin compaction and decompaction. It is a Megadalton, multisubunit protein complex, which includes ATP-dependent remodeling enzymes CHD3/4, pRB-associated proteins RBBP4 and RBBP7, CpG-binding proteins MBD2/3, the GATAD2a and/or GATAD2b, specific DNA-binding proteins MTA1/2/3, and histone deacetylase HDAC1/2 [25, 26]. However, several subunits (e.g., MBD2/MBD3, CHD3/CHD4) of this complex are mutually exclusive, and it remains elusive how and when these subunits associate with the NuRD complex. Deep proteomics data analysis revealed that PTK6-phosphorylated-SMAD4 forms a complex with the NuRD complex, which includes CHD4, MBD3, HDAC1/2, GATAD2b, RBBP4, RBBP7, MTA1, and MTA2; it does not include MTA3 [19].

A recent discovery demonstrated that SMADs can directly form a complex with histone deacetylase and suppress gene expression. HDAC8, a class I HDAC, forms a heterotrimer complex with SMAD3/4 and occupies the *SIRT7* promoter to deacetylate H4 via local chromatin remodeling. This results in the suppression of *SIRT7* gene expression [27]. It is worth mentioning that the inhibition of histone deacetylase activity of HDAC8 attenuates the deacetylase activity of the SIRT7-SMAD4 axis, resulting in the inhibition of lung metastasis and the improvement of the efficacy of chemotherapy in breast cancer [27]. Moreover, upon activation of the TGF-β/SMADs signaling, SMAD3 interacts with HDAC4/5 (class IIa HDACs) via the MH2 domain and forms SMAD3-HDAC4/5 and deacetylates H4 at the *osteocalcin* promoter, resulting in transcriptional repression of *Runx2* [28], which is required for osteoblasts differentiation and bone formation.

Notably, drug exposure (e.g., cocaine) induces SMAD3 interaction with BRG1 [29], an ATPase subunit of the SWI/SNF chromatin-remodeling complex, and negatively regulates cell proliferation and suppresses tumor pathogenesis. It has been reported that BRG1 incorporates into the transcriptional complexes of SMAD2/3-SMAD4 and modulates gene expression; however, the role of the SWI/SNF-BRG1-SMADs complex in gene regulation during drug addiction is unknown. It is also reported that the TGF-β pathway uses p300/CBP, a histone acetylase, for transcriptional activation of target genes. The SAD domain of SMAD4 directly binds with p300/CBP and recruits to the SMAD complex for transcriptional activation [30, 31]. Recently, it has been shown that in healthy cells, SMAD nuclear-interacting protein 1 (SNIP1) forms a complex with SMAD4 and inhibits the acetyltransferase activity of SMAD4/p300, resulting in suppression of cell migration-related genes. However, in cancer cells, DPF3a—a short isoform of DPF3 which is a component of the SWI/SNF chromatin-remodeling complex—binds to SNIP1 and releases it from SMAD4/p300 histone acetylase complex. This leads to enhanced chromatin acetylation and subsequent expression of cell migration-related genes, which eventually promote metastasis [32].

Besides being writers and erasers of histone code, SMADs proteins are also associated with the regulation of histone readers. For example, a well-characterized histone reader TRIM33, also known as TIF1γ, was initially discovered as a transcription corepressor [33]; however, it can also act as a promoter of transcription by recruiting transcription elongation factors p-TEFb and FACT [34]. TRIM33 binds to the activated SMAD2/3 complex in competition with SMAD4 in response to TGF-β. TRIM33-SMAD2/3 complex mediates erythroid differentiation and, on the other hand, the SMAD2/3-SMAD4 complex inhibits cell proliferation in response to TGF-β in hematopoietic, mesenchymal, and epithelial cell types [35]. The PHD finger-bromodomain of TRIM33 specifically recognizes and binds unmodified K4 and R2 and acetylates at least 2 lysines of the histone H3 tails. Additionally, Xi et al. reported that nodal-activated TGF-β signaling induces SMAD4-SMAD2/3 and TRIM33-SMAD2/3 complex formation and prompts differentiation of mammalian embryonic stem cells. The PHD finger-bromodomain of TRIM33 facilitates TRIM33-SMAD2/3 binding to the H3K9me3 and H3K18ac on the promoters of *Gsc* and *Mixl*, resulting in the displacement of the chromatin-compacting factor HP1γ, which allows the SMAD4-SMAD2/3 complex to recruit Pol II, poising chromatin in the active state during embryonic stem cells differentiation [36].

In sum, the elegant simplicity of this core signal transduction pathway sharply contrasts with the intricacy of the elicited biological responses. Seemingly straightforward, TGF-β/SMAD signaling elicits a dizzying array of biological responses by interacting with transcription factors, coactivators, chromatin remodelers, and histone modifiers.

## Hippo signaling pathway in chromatin regulation during carcinogenesis

**Fig. 3:**
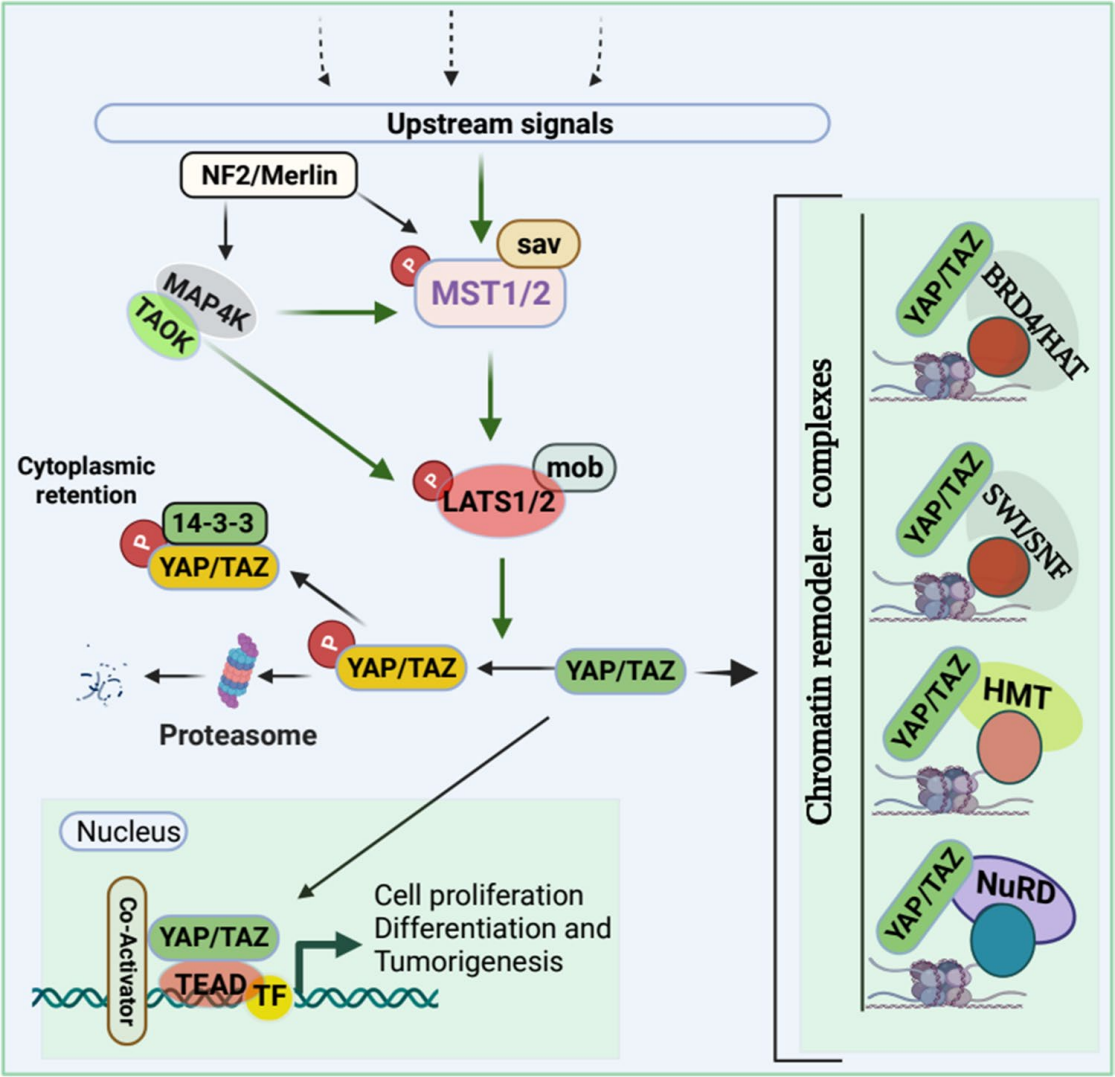
Schematic of the Hippo signaling pathway in the regulation of chromatin remodeler complexes. Several upstream stimuli/signals can activate Hippo signaling through the phosphorylation of MST1/MST2 and subsequent phosphorylation of LATS1/LATS2 kinases leading to YAP/TAZ phosphorylation resulting in proteasomal degradation or cytoplasmic retention of YAP/TAZ via 14–3-3 protein. Additionally, Neurofibromatosis 2 (NF2) along with MAP4k and TAOK can also activate Hippo signaling without MST1/MST2 phosphorylation. However, when the Hippo pathway is off, YAP/TAZ will not be phosphorylated, thus, nonphosphorylated YAP/TAZ translocates into the nucleus and form complexes with transcription factors, co-activators as well as different chromatic remodeler complexes to facilitate the transcription target genes. Dotted lines indicate upstream signals

The Hippo pathway is highly conserved across vertebrates and plays a critical role in organogenesis and homeostasis through the precise controlling of cell proliferation, apoptosis, differentiation, metabolism, and determination of cellular fate and organ size [37–40]. The Hippo signaling pathway largely depends on mammalian effectors Yes-associated protein (YAP) and transcriptional activator with PDZ binding motif (TAZ) for target genes regulation and subsequent biological processes. In mammals, Hippo signaling is composed of (1) serine/threonine kinase cascade Mammalian STE20-Like Protein Kinase 1 and 2 (MST1 and MST2), (2) adaptor protein Sav family WW domain-containing protein 1 (SAV1), (3) large tumor suppressor kinase 1/2 (LATS1/2), (4) adaptor proteins MOB1A/1B, and (5) the transcription coactivators YAP/TAZ [41]. In parallel with MST1/2, MAP4K and TAOK kinases directly phosphorylate LATS1/2 to activate Hippo signaling [42, 43]. Additionally, tumor suppressor neurofibromin 2 (NF2), also known as Merlin, potentially activates LATS1/2 in MAP4K- and TAOK-dependent manner to activate the Hippo pathway, resulting in the inhibition of YAP and TAZ activity [43]. Activated Hippo signaling promotes phosphorylation of YAP/TAZ, which results in either 14–3-3 protein-dependent cytoplasmic retention [44] or proteasomal degradation, [45] which inhibits YAP and TAZ activity (Fig. 3).

Dysregulation of the Hippo pathway is associated with human pathogenesis, including tumor development, progression, and metastasis [41]. YAP and TAZ are frequently amplified in cancers and associated with hyperproliferation, cancer-cell maintenance, cellular invasion, metastasis, and chemoresistance. Through a comprehensive analysis of The Cancer Genome Atlas, scientists found that *YAP* and *TAZ* were the most frequently amplified genes among the 19 core genes of the Hippo pathway in squamous cell cancers. Moreover, *YAP* and *TAZ* were mutually amplified in cervical squamous cell carcinoma and head and neck squamous cell carcinoma [6]. It is worth noting, although the Hippo pathway is one of the most altered pathways in cancer [46], mutation frequencies in this signal transduction pathway are low [6, 47]. Recent progress suggests that besides *YAP/TAZ/TEAD* amplifications, crosstalk with chromatin-remodeler complexes strongly associates with tumorigenesis, metastasis, chemoresistance, and poor prognosis.

Coactivators YAP/TAZ bind to DNA-binding factors of the TEAD transcription factor family (TEAD1–4) to regulate the transcription of target genes [38]. YAP/TAZ also interacts with several other transcription factors, including SMAD2/3 [48], RUNT-related transcription factors (RUNX1 and RUNX2), and p73 [49], and forms a complex with T-box transcription factor 5 (TBX5) and β-catenin [50] to regulate the transcription of target genes and to promote cell proliferation, survival, growth, and migration [51]. For example, YAP recruits Trithorax-related histone methyltransferase (HMT) complex via nuclear receptor coactivator 6 (Ncoa6), a subunit of the HMT complex, to facilitate H3K4 methylation, which results in transcriptional activation of Hippo target genes to promote cell proliferation and cell survival [50].

Besides transcriptional activation, YAP can also act as a transcriptional repressor in association with chromatin remodeler and histone deacetylase complexes to attenuate proapoptotic and cellular growth. YAP interacts with CHD4, a component of the NuRD complex, and recruits the NuRD complex to the promoter to suppress the transcription of NR4A1 [52]. NR4A1 (also known as Nur77, or TR3, or NGFIB) is an orphan nuclear receptor that plays a significant role in proapoptotic function by binding with Bcl-2 [53]. Ectopic expression of NR4A1 slowed cell proliferation, reduced the capability to form colonies in several cancer cells, and inhibited tumor growth in mouse xenograft models [52]. Moreover, the SWI/SNF complex is essential for TAZ to regulate target genes expression. TAZ interacts with BRM, the catalytic subunits of the SWI/SNF complex, and recruits to the promoter of *CTGF*, a bona fide target gene. This results in an induction of this gene in MCF10A cells [54]. It is worth noting that copy-number amplification of TAZ is observed in more than 44% of triple-negative breast cancers (TNBC), whereas only 10% (luminal A) and 20% (luminal B) in estrogen-positive breast tumors. In accordance with copy-number amplification, TAZ protein levels are higher in TNBC than estrogen receptor-positive breast tumors [54]; this suggests a potential role for TAZ in TNBC heterogeneity and metastasis.

Recently, it has been reported that tumor suppressor FAT1 inhibits cell proliferation [55], migration, EMT, and metastasis [56] by activating the Hippo pathway. FAT1 promotes TAOKs-mediated phosphorylation of MST1, leading to the activation of the Hippo kinase cascade, resulting in YAP inactivation [57]. In contrast, mutant-FAT1 alters the Hippo pathway by modulating the core Hippo-kinase signalosome. This results in increased nuclear localization of YAP/TAZ in head and neck squamous cell carcinoma [58], thereby YAP1 transcriptional program activation in the promotion of cancer growth and progression [59]. YAP/TAZ is a chromatin-binding protein that physically interacts with coactivator bromodomain-containing protein 4 (BRD4), a chromatin-binding protein, and dictates the genome-wide association of BRD4 with chromatin to recruit RNA polymerase II and boost the expression of growth-regulating genes [60]. YAP1/TAZ recruited BRD4, a histone acetyltransferase, acetylates histones H3 and H4 specifically histone mark H3K122ac to enhance the transcriptional activity of the YAP1/TAZ target genes to support the growth of cancer cells [60, 61]. It is also reported that the AAA ATPase and bromodomain factor (ATAD2), a transcriptional coactivator, guides proteins toward acetylated histones to regulate chromatin dynamics associated with YAP1 transcriptional activation in head and neck squamous cell carcinoma [58]. Moreover, TAZ-CAMTA1 and YAP-TFE3 (TAZ fused to the protein CAMTA1 and YAP fused to the protein TFE3) bind with the chromatin remodeler and histone acetyltransferase ATAC complex to activate TAZ- and YAP-regulated transcription program to drive uncontrolled, cancerous growth.

In summary, YAP/TAZ, the downstream effector of the Hippo pathway, encourages wider chromatin accessibility by interacting with chromatin-remodeler complexes. In association with chromatin-remodeler complexes, YAP/TAZ activates transcriptional programs to promote cell proliferation, EMT, migration, and tumor metastasis; thus, targeting YAP/TAZ, which highlights it as a promising therapeutic option for several cancers.

## Wnt signaling pathway in chromatin regulation during carcinogenesis

**Fig. 4:**
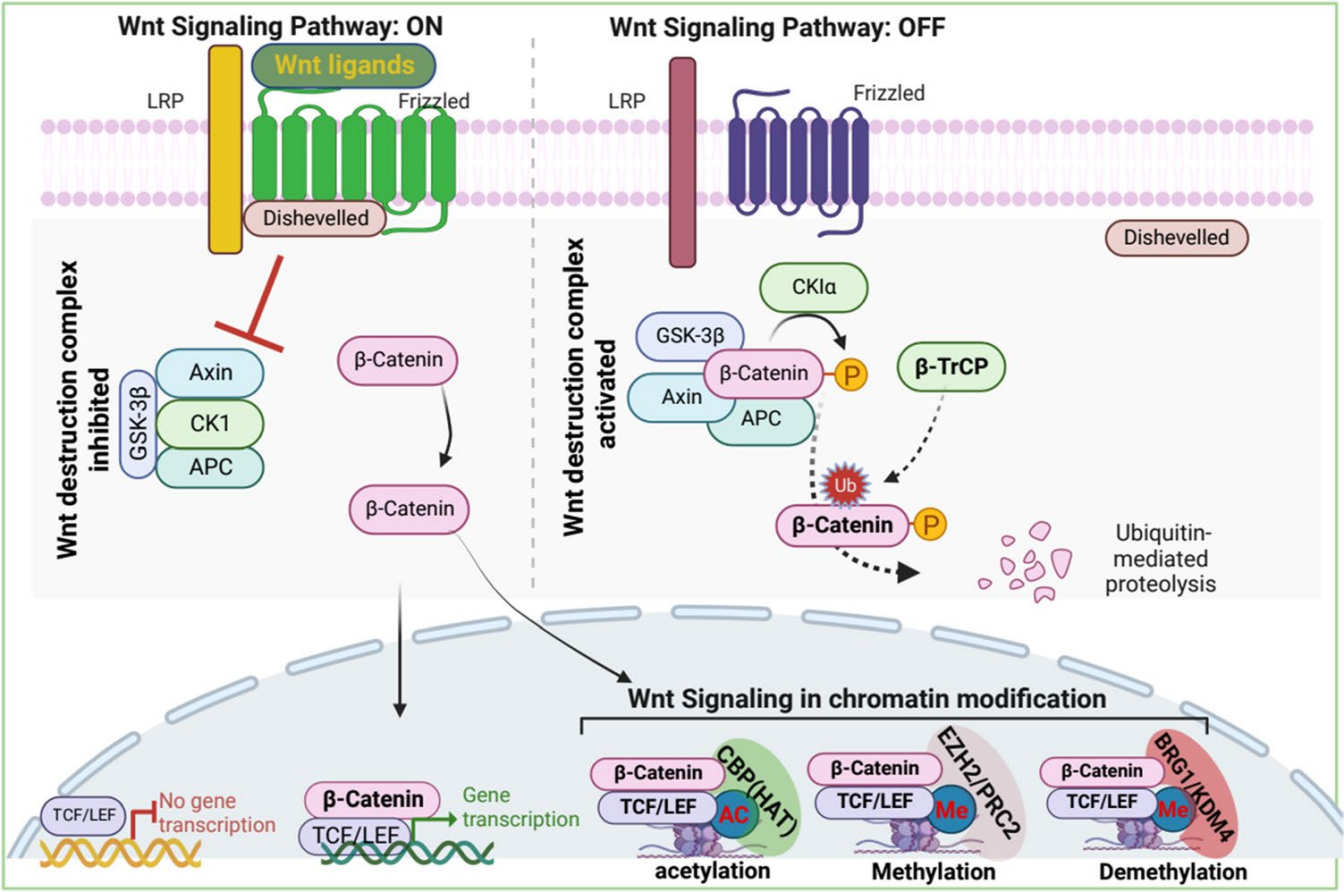
Activation of Wnt signaling pathway promotes β-Catenin–mediated chromatin regulation. In the absence of Wnt ligands, activated wnt-destruction-complex phosphorylates β-Catenin and promotes ubiquitin-mediated proteolysis of β-Catenin. However, wnt-ligands dependent activation inhibits wnt-destruction-complex, resulting in the stabilization of β-Catenin. The stabilized β-Catenin translocates into the nucleus and the accumulation of nuclear β-Catenin facilitates the transcription of its target genes in association with several chromatin remodeler complexes. The dotted lines indicate further experimental validation is required

The Wnt signaling pathway is one of the most extensively studied signaling pathways. Wnt plays a critical role in embryogenesis and adult homeostasis. However, aberrant activation of this pathway is implicated in tumorigenesis and metastasis. This signaling pathway is highly conserved and can be activated through canonical or noncanonical mechanisms. In canonical Wnt signaling, intracellular Wnt ligands (e.g., Wnt3a and Wnt1) bind to Frizzled receptors (FZD) and low-density lipoprotein receptor-related proteins (LRP5/6). Subsequently, CK1α and glycogen synthase kinase 3β [GSK-3β] phosphorylate LRP5/6 of the FZD-LRP5/6 complex; this triggers the recruitment of Dishevelled (Dvl) proteins to the plasma membrane [62]. Dvl and FZD-LRP5/6 form a cytoplasmic signalosome that is stably polymerized [63]. The Dvl-containing polymerized signalosome directly inhibits GSK-3β [63], thereby destabilizing the β-catenin destruction complex. (The destruction complex is mainly composed of adenomatous polyposis coli [APC], axis inhibition protein [AXIN], GSK3, and casein kinase 1 [CK1] [62].) Destabilization of the β-catenin destruction complex results in β-catenin accumulation and nuclear translocation and signaling. However, in the absence of Wnt ligands, GSK-3β phosphorylates β-catenin followed by β-transducin repeat-containing protein (β-TrCP) E3 ligase mediated ubiquitination and proteasomal degradation [64–66], ultimately leading to inhibition of Wnt signaling and expression of Wnt target genes (Fig. 4).

The noncanonical Wnt signaling pathways are diverse and less distinct, often defined as β-catenin–independent Wnt- or FZD-initiated signaling [67] (reviewed in Anastas and Moon). The noncanonical Wnt signaling transduction pathways include several signaling cascades, such as Wnt-PCP (planar cell polarity) signaling, Wnt-cGMP/Ca^2+^ signaling, Wnt-ROR2 signaling, Wnt-RAP1 signaling, Wnt-PKA signaling, Wnt-GSK3-microtubule (MT) signaling, Wnt-aPKC signaling, and Wnt-mTOR signaling [68]. The implication of the noncanonical Wnt pathway in cancers is largely unknown. Studies have shown that noncanonical Wnt signaling is involved in several physiological processes, including stem cell maintenance [69], tumor progression [70], and tumor suppression [71]. Although the correlation between noncanonical Wnt pathways and cancer is undisputed, this review focuses on canonical Wnt signaling and the epigenetic modifiers that are exploited during tumorigenesis and metastasis.

The Wnt pathway modulates epigenetics to promote cancer initiation and progression. The downstream effector of Wnt signaling, β-catenin, interacts with DNA methyltransferase I (Dnmt1); this interaction stabilizes both proteins. This Wnt/β-catenin/Dnmt1 complex alters DNA methylation patterns on a specific locus and drives tissue differentiation [72]. In colon adenocarcinoma tissues, Dnmt1 hypermethylates the promoter of *NHERF1*, resulting in epigenetic silencing of *NHERF1*. This silencing has been associated with an EMT phenotype in colon cancer [73]. Moreover, β-catenin recruits EZH2, an enzymatic catalytic subunit of the polycomb repressive complex 2 (PRC2), which trimethylates Lys-27 in histone 3 (H3K27me3) to suppress genes expression through polymerase-associated factor 1 (PAF1). This enhances the transactivation of Wnt target genes in cancers [74]. Overall, β-catenin–mediated transcriptional regulation is largely dependent on the interacting partners and the place of recruitment; thus, recruitment is crucial in determining whether it would function as a tumor suppressor or oncogene.β-catenin directly interacts with acetyltransferases p300/CBP to modify the structure of chromatin. This chromatin remodeling results in acetylation and reorganization of chromatin, thereby allowing recruitment of the transcription machinery to promoters of Wnt target genes [75]. The C-terminal domain of β-catenin acts as a scaffold and interacts with diverse factors, including BRG1, ISWI, HMTs, the Mediator component MED12, and PAF1, to modify the histones and to rearrange histone structure, thus inducing rapid gene expression [74]. Moreover, β-catenin interacts with lysine demethylases (KDMs) and demethylates the repressive marks on histones. KDM4D interacts with β-catenin and erases methyl groups from H3K9me3, a marker of transcriptional suppression, to augment gene expression of Wnt/β-catenin target genes [76]. In stem cells from human colorectal cancer, the β-catenin/Tcf complex recruits KDM3A and KDM3B to demethylate H3K9me2 and promotes MLL1-mediated H3K4 methylation. In turn, BCL9/PYGO is recruited to the chromatin, which leads to the transcription of Wnt target genes [77, 78].

In the absence of Wnt signaling, LEF1 interacts with HDAC1 and recruits the NuRD complex to repress the transcription of Wnt target genes. However, when Wnt signaling is activated, β-catenin removes HDAC1 from the HDAC1-LEF1 complex and attenuates the deacetylase activity of HDAC1. β-catenin then forms the dimeric β-catenin-LEF1 complex that activates the transcription of Wnt target genes [79]. Although β-catenin–mediated attenuation of HDAC1 activity is important for the transcriptional activity of β-catenin-LEF1 complex, how β-catenin enzymatically inactivates HDAC1 remains unclear. It has been reported that β-catenin competes with HDAC1/2 for its main transitional activator, T cell factor 4 (TCF4), to form the β-catenin-TCF4 complex, which activates gene expression and is essential for intestinal homeostasis and tumorigenesis [80, 81].

The Wnt signaling downstream effector β-catenin acts as a molecular switch to regulate global gene activation by interacting with transcription factors and chromatin-remodeler complexes. For instance, the chromatin organizer special AT-rich binding protein 1 (SATB1) recruits β-catenin and p300 acetyltransferase to induce *GATA-3* expression during helper T cell differentiation [82]. The precise spatiotemporal regulation of gene expression during tumorigenesis, and metastasis is a critical event that depends on the Wnt-induced rearrangement of chromatin to poise genes for gene expression.

## Notch signaling pathway in chromatin regulation during carcinogenesis

**Fig. 5:**
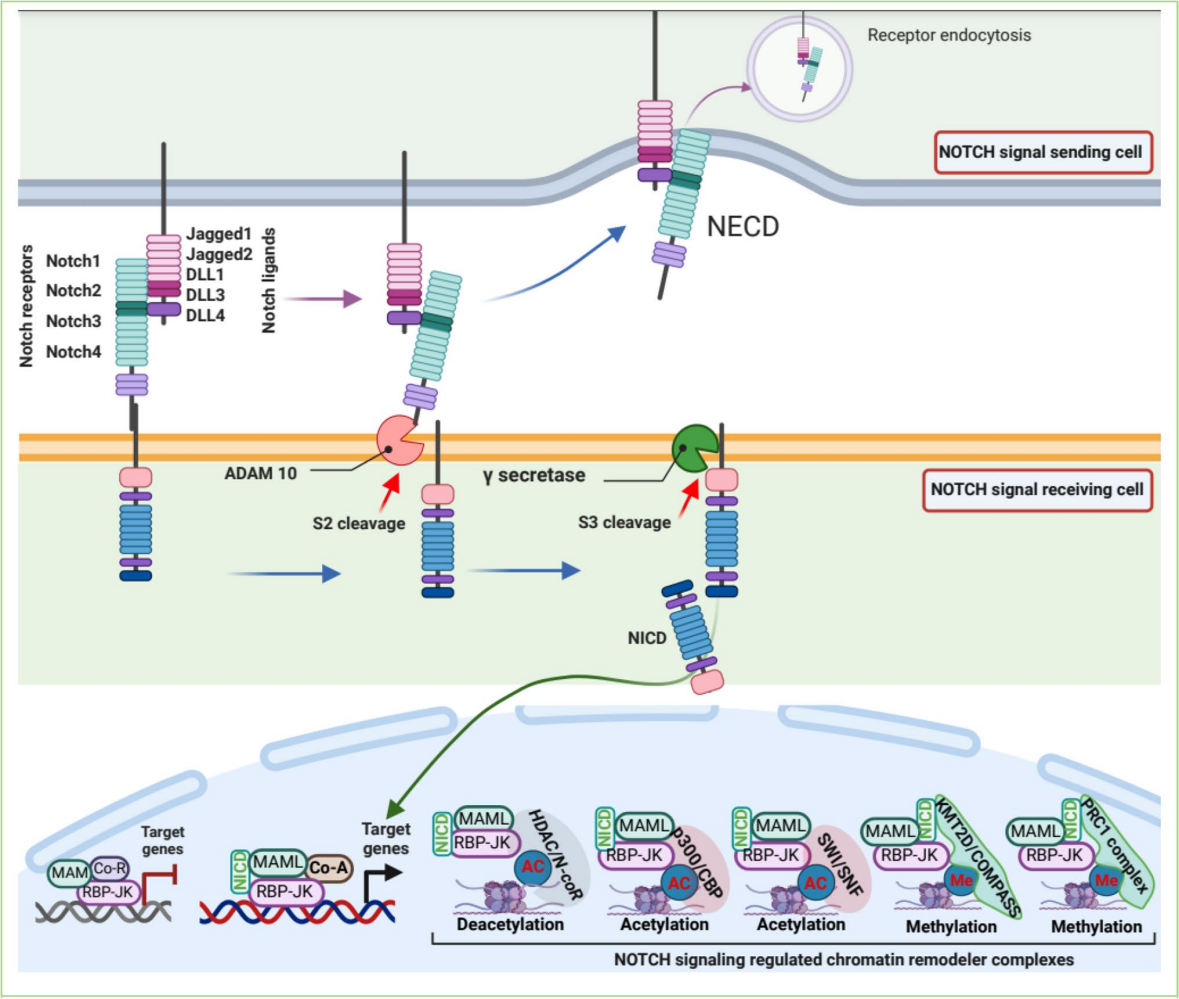
Notch signaling pathway in chromatin regulation. The binding of the notch-ligand from the signaling cells to the notch-receptor of signal-receiving cells promotes the proteolytic cleavages of the notch-receptor. Metalloprotease ADAM10 catalyzes the S2 cleavage followed by γ-secretase dependent catalysis of S3 cleavage, resulting in release of the Notch intracellular domain (NICD). Released NICD translocates into the nucleus, where it interacts with the co-activators and DNA-binding proteins and chromatin modifying complexes for regulating transcription

The Notch signaling pathway is a conserved master intracellular pathway that regulates diverse developmental processes, including organ formation, tissue function, and tissue homeostasis through cell-to-cell communication [83, 84]. In humans, Notch receptors (4 receptors: NOTCH1, 2, 3, and 4) and their ligands (Delta-like [DLL1, 3, and 4] and Jagged 1 and 2) are transmembrane proteins; thus, the Notch signaling pathway is restricted to adjacent cells [85]. In canonical Notch signaling, when a Notch ligand-expressing cell (signaling cell) directly binds with a Notch receptor-expressing cell (receiving cell), the ligand-receptor interaction triggers the Notch signaling cascade. The ligand-receptor interaction promotes 2 proteolytic cleavage events of the Notch receptor by the protease ADAM10 or by TACE, which is subsequently cleaved by γ-secretase to release of Notch Intra Cellular Domain (NICD) [85]. The released NICD translocates into the nucleus and coordinates with the coactivator Mastermind (Mam) or the DNA-binding protein CSL (CBF1 Suppressor of Hairless Lag1, also known as RBP-JK) to regulate the transcription of Notch target genes [84, 86] (Fig. 5). Although the framework of the Notch signal is remarkably simple, Notch signaling is involved in a variety of cellular processes. Given this, defective Notch signaling has been implicated in a variety of human malignancies, including cancer progression and metastasis.

The dysregulation of Notch signaling can be involved in proliferation and growth arrest during differentiation, either survival or death pathways, and is largely dependent on the physiological conditions and protein–protein interactions; thus, it is not surprising that Notch signaling shows mixed, and contradicting, effects across tumor types. A growing body of emerging evidence supports the idea that Notch signaling is a major player in tumor initiation and progression in different cancers, including small cell lung cancers [87], peripheral T cell lymphoma [88, 89], and osteosarcoma [90]. Moreover, NOTCH signaling promotes EMT in BRCA1-defective conditions, resulting in initiation of TNBC [91]. However, conditional gene deletion or overexpression of NOTCH2 can suppress tumor growth cooperation with p53 glioma mouse models of human brain tumors [92]; this indicates that Notch signaling may play both an oncogenic and a tumor-suppressive role in human cancers.

In recent years, a growing body of the literature suggests that the interaction of effector proteins (NICD, RBP-J, and MAML) with chromatin-remodeler complexes is responsible for the activation of the NOTCH-targeted genes [93, 94]. It has been reported that NOTCH effector bifunctional protein RBP-JK recruits the histone deacetylase activity containing corepressor N-CoR complex to deacetylate histone tails and repress transcriptional activity. The RBP-JK–mediated suppression of gene expression is therefore reversed in the activation of NOTCH signaling [95]. Nonetheless, activation of Notch signaling promotes NICD interaction with KMT2D, a lysine methyltransferases of COMPASS complexes (complex of proteins associating with Set1), resulting in the displacement of the N-CoR complex, which allows recruitment of MAML and the HAT p300 [96, 97]. Moreover, p300 markedly enhances transcription from chromatin templates in conjunction with MAML in in vitro settings [94]. The Notch transcription complex (NICD, RBP-JK, and MAML) recruits p300 [98], which influences H3K27 acetylation marks [99] and the recruitment of KMT2D, and promotes H3K4 methylation [100], which in turn promotes the transcription of *Myc*; this might be an important event in several human tumors, including T-ALL [101], breast cancer [102], and mantle cell and marginal zone lymphoma [103, 104].

Chromatin remodeling mediated by Notch signaling, and the subsequent changes in gene expression caused by the chromatin remodeling, remain contentious. Genetics and phenotypic studies have shown that activated Notch and its binding partner, RBP-JK, recruit the SWI/SNF chromatin-remodeling complex, which positively regulates Notch-targeted transcription activity [105, 106]. However, during the differentiation of retinal progenitor cells into different retinal cell types, BRM, an ATPase subunit of the SWI/SNF chromatin-remodeling complex, interacts with RBP-JK and prevents NICD-RBP-JK complex formation. Concomitantly, RBP-JK recruits the SWI/SNF complex to the promoter of *Hes1*/*Hes5* to suppress Notch-mediated transcription [107]. Recently, it was reported that NICD-RBP-JK-MAML containing Notch-repressive complex (NRC), recruits the polycomb repressive complex 1 (PRC1) to repress *MAD4*, a MYC repressor, resulting in the elevation of MYC expression and oncogenic functions [108].

Notch signaling is activated through cell-to-cell contact, and cancer cells take advantage of elevated expression of Notch ligands for the activation of Notch signaling in endothelial cells to activate angiogenesis, an indispensable process for sustainable tumor growth and progression [109–112]. Lymphoma cells express FGF4 to activate FGFR1 in neighboring endothelial cells to upregulate the Notch ligand Jag1. In turn, upregulated Jag1 in endothelial cells activates Notch signaling in lymphoma cells to induce *Hey1* expression, which in turn makes lymphoma cells aggressive and chemoresistance [113]. Under hypoxia, hypoxia-inducible factor 1 (HIF-1) recruits the NTC complex to promote the expression of SNAI1, resulting in EMT, tumor cell migration, and invasion [114] in several cancers, including oral squamous cell carcinoma [115], bladder [116], and pancreatic cancers [117]. Hence, the deceptively simple Notch signaling pathway plays an extremely dynamic role by interacting with several coactivators including chromatin-remodeler complexes HAT, NuRD, SWI/SNF, PRC1, and N-CoR in tumor suppression, progression, and metastasis.

Thus, it is conceivable that the Notch signaling pathway serves as a platform for transcription activators and corepressors to regulate target genes in accordance with the physiological condition. This suggests an extremely dynamic, multifunctional role for Notch signaling in oncogenic processes. In the near future, we expect more scientific evidence to delineate the diverse roles of Notch signaling in different physiological conditions; this information is essential if we hope to develop therapeutic strategies to modulate Notch signaling.

## PI3K/AKT signaling in chromatin regulation during carcinogenesis

**Fig. 6:**
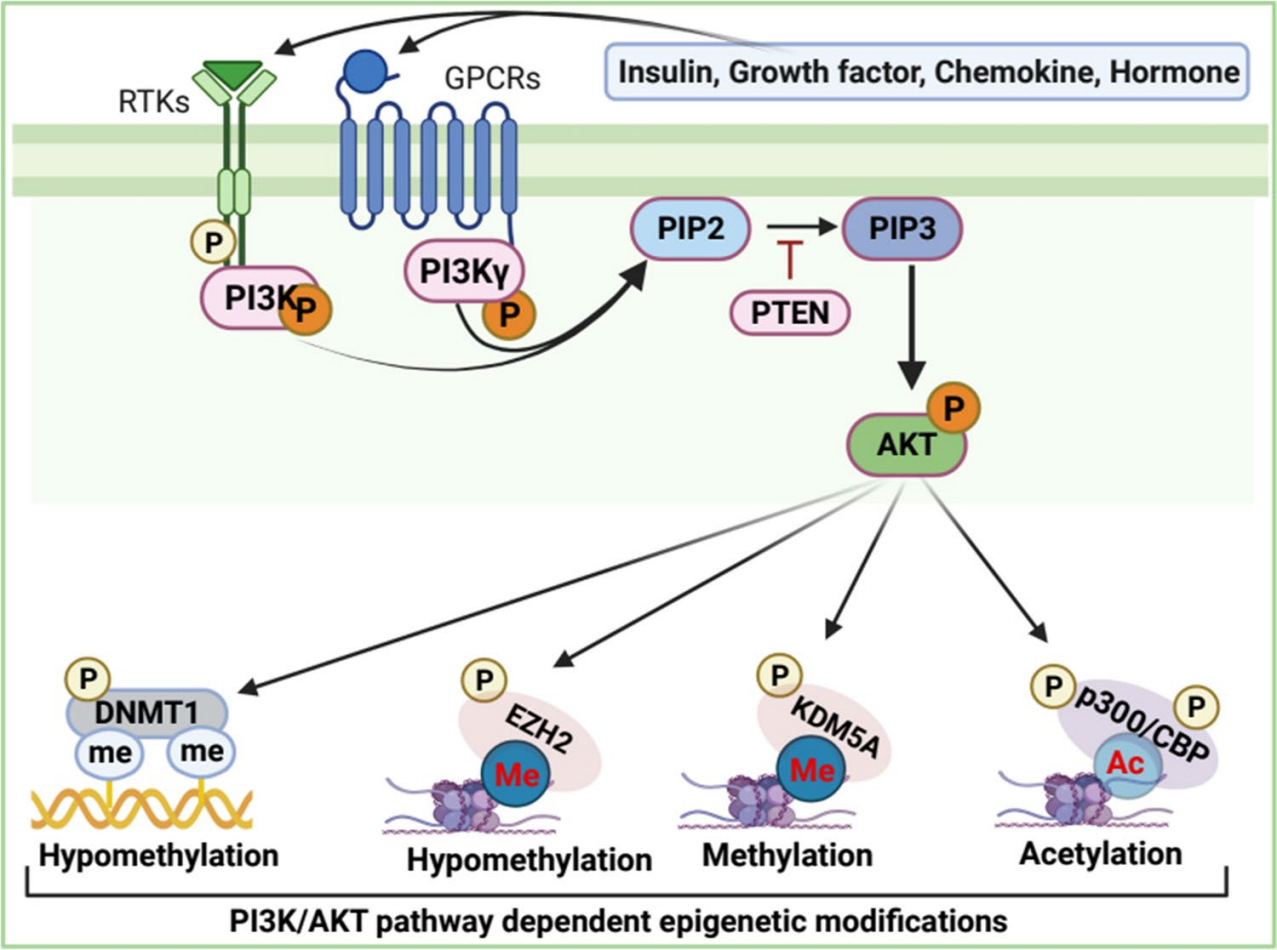
PI3K/AKT signaling dependent epigenetic regulation. In response to extracellular stimuli, membrane-bound receptor RTKs/GPCRs gets activated leading to phosphorylation of PI3K which catalyzes the phosphorylation of PIP2 to produce PIP3 resulting in the activation of Akt. Activated AKT phosphorylates a variety of substrates, including chromatin remodeler complexes, to regulate epigenetic gene regulation

The PI3K/AKT/mTOR signal transduction pathway is one of the most critical pathways as it is involved in numerous biological processes, including cell proliferation, migration, adhesion, invasion, metabolism, and survival [118]. Aberrant activation of PI3K/AKT/mTOR signaling is frequently observed in most human cancers that modulate apoptosis, autophagy, EMT, tumorigenesis, metastasis, and chemoresistance [118–120]. Oncogenic PI3K/AKT/mTOR signaling is extensively studied and reviewed elsewhere [121]. Briefly, the activation of this signaling initiates from cell-surface receptor tyrosine kinases (RTKs) or G-protein-coupled receptors, leading to plasma membrane recruitment of the lipid kinase PI3Ks (composed of catalytic subunits p110α and regulatory subunits p85α) which catalyzes the phosphorylation of PtdIns 4,5-bisphoshate (PIP2) to produce phosphatidylinositol 3,4,5-trisphosphate (PIP3), which in turn activates the downstream protein serine–threonine kinase AKT (also known as protein kinase B or PKB) (Fig. 6). Activated AKT phosphorylates a diverse array of downstream substrates, including Bcl-2 antagonist of cell death, glycogen synthase kinase-3, forkhead transcription factors, and mTOR complex (mTORC1) to regulate a variety of cellular functions, including cell proliferation, growth, survival, migration, and anabolic biosynthesis [122, 123]. In contrast, phosphatase and tensin homologue (PTEN) catalyzes the dephosphorylation of PtdIns(3,4,5)P3 to regenerate PtdIns(4,5)P2, resulting in the inactivation of PI3K/AKT signaling [121]. Besides growth factor-dependent activation of PI3K/AKT signaling, this signaling frequently activates growth factor independently in human cancers [121]. Dysfunction of PI3K/AKT signaling is also associated with diverse pathological settings, including cancers [124]. A plethora of evidence indicates that PI3K/AKT signaling promotes oncogenicity by directly or indirectly modulating epigenetic modifiers for epigenetic reprogramming in cancers [125–127].

Epigenetic silencing of tumor suppressor genes or activation of oncogenes is frequently observed during tumorigenesis. DNA and histone modifications can lead to the epigenetic silencing of genes. Hypermethylation of DNA is often associated with gene silencing. For example, promoter methylation causes the suppression of the retinoblastoma gene (Rb) in retinoblastoma-tumors [128]. On the contrary, activated PI3K/AKT signaling stabilizes DNA methyltransferase 1 (DNMT1) through AKT-mediated phosphorylation, resulting in increased DNMT1 methyltransferase activity [129]. As such, PI3K/AKT signaling reduces global genomic DNA methylation and promotes transcriptional activation of specific gene loci in breast cancer cells [125]. It is reported that PI3K/AKT signaling regulates DNMT3 activity in both GSK3α/β-dependent or independent manner to regulate locus-specific DNA hypomethylation for specific genes transcription [130]. Thus, the frequent imbalance of DNA methylation and demethylation during tumorigenesis could be associated with the aberrant activation of the PI3K/AKT signaling pathway.

Mounting evidence suggests that PI3K/AKT signaling-mediated chromatin regulation (histone modifications) by chromatin-remodeler complexes induces transcriptional activation to promote tumorigenesis. AKT phosphorylates methyltransferase EZH2, a member of PRCs, that trimethylates promoter-associated histone H3 Lys27 (H3K27me3) to suppress transcription [131]. Activated AKT phosphorylates serine 21 of EZH2, resulting in the reduction of EZH2 methyltransferase activity and binding to histone H3, which reduces H3K27 trimethylation [131], hinting that transcription activation may contribute to the oncogenesis. Moreover, phosphorylated EZH2 acts as a transcriptional coactivator in association with the androgen receptor and other transcription factors to promote oncogenesis [132]. AKT phosphorylates H3K4-demethylase-KDM5A and regulates its subcellular localization and genome occupancy, resulting in increased H3K4 trimethylation, prompting upregulation of a set of genes associated with cell-cycle in breast cancer [133].

PI3K/AKT signaling regulates p300/CBP-mediated histone acetylation of lysine residues to activate transcriptional activity has been implicated in tumorigenesis and metastasis in different cancer types [134]. Upon activation of PI3K/AKT signaling, activated AKT phosphorylates histone acetyltransferase (HAT) p300/CBP, acetylate hundreds of histone/non-histone substrates [135], resulting in the stimulation of acetyltransferase activity of p300/CBP complex, leading to recruitment of basal transcriptional machinery for gene expression [136]. It has also been reported that the activation of the Ras-PI3K-AKT pathway promotes MDM2-dependent proteasomal degradation of p300/CBP, resulting in the reduction of H3K56ac, which is associated with tumor cells proliferation and migration [137]. Although high H3K56Ac is proportional to tumor grade and tumorigenicity, H3K56Ac is not associated with breast cancer-cell proliferation [138]. P300/CBP also acetylates H3K18; however, AKT-dependent phosphorylation of CBP at Thr187, but not p300, changes its affinity for H3, resulting in reduced H3K18ac, which promotes oncogenic activities and tumor progression [139].

AKT promotes PRC1 complex-mediated H2A ubiquitination to repress chromatin at specific genomic loci for transcriptional inactivation. Specifically, E3 ubiquitin ligase RING1A containing PRC1 complex monoubiquitinates nucleosomal histone H2A at lysine 119 and subsequently recruits PRC2, which catalyzes the addition of methyl groups to histone H3 at lysine 27 (H3K27me3). This contributes to polycomb-complex dependent epigenetic gene silencing [140]. AKT-mediated phosphorylation of Bmi1, a transcriptional silencer of PRC1 complex, triggers its removal from the Ink4a-Arf locus that encodes the p16INK4A and p19ARF tumor suppressors resulting in decreased H2A ubiquitylation, leading to accumulation of p16 and p19 result in inhibition of cell proliferation and induction of cellular senescence of cancer cells [141]. Although p16 and p19 are antiproliferation proteins that are typically incompatible with the oncogenic growth yet enriched in PI3K/AKT signaling-driven cancer cells. Oncogenic signaling-induced senescence observed in cancer cells may be driven by the aberrant activation of PI3K/AKT signaling and subsequent phosphorylation of Bmi1, a transcriptional silencer of PRC1 complex.

Overall, the PI3K/AKT signal transduction pathway has emerged as a critical signal transducer that is most frequently dysregulated in virtually all solid tumors and hematological malignancies. The downstream effector of this signaling pathway, AKT, phosphorylates over 200 substrates [122, 142] and modulates epigenetic reprogramming, which most likely contributes to the diverse cellular functions of PI3K/AKT signaling.

## Future perspectives and concluding remark

Signal transduction pathways are a critical means of cellular communication; these pathways allow cells to perceive extracellular signals/stimuli and transmit those signals into precise cellular functions, thus maintaining cellular and organismal homeostasis. Understanding the detailed molecular mechanisms that contribute to cell signal transmission from the cell membrane to the nucleus and that regulate chromatin-remodeler complexes, transcriptions activators, and cofactors to control gene expression is critical if we hope to understand disease mechanisms. For example, HDAC1 and HDAC2 deacetylate over 50% of global histones acetylation marks and take part in 3 different histone deacetylase complexes: SIN3/HDAC, NuRD, and CoREST. How stimuli-dependent and context-dependent cellular signaling contributes to the distribution of HDAC1 and HDAC2 in those chromatin-remodeler complexes in normal and diseased conditions is critical to devise therapeutic interventions in human cancers.

A subgroup of tyrosine kinases, known as non-receptor tyrosine kinases, relay intracellular signals and is indispensable in cell signaling [143]. Elevated expression and aberrant activation of nRTKs often dysregulate signaling pathways to promote human pathogenesis, including cancer. For example, SRC promotes TGF-β signal-mediated tumor growth and metastasis [144] and PEAK1-mediated dysregulation of TGF-β signaling induces EMT and metastasis in breast cancer cells [18]. A recent study from the Washburn lab reported that PTK6, a nRTK, catalyzes the phosphorylation of tyrosine 352 and 412 of SMAD4 [19] and alters TGF-β/SMAD signaling, thereby enhancing the metastatic potential of breast cancer cells. The extensive proteomics data from the same study revealed that PTK6/phosphorylated-SMAD4 interacts with the core subunits of chromatin remodeling and histone deacetylase NuRD complex and forms a PTK6-_phos_SMAD4-NuRD complex. The above-mentioned evidence suggests that nRTKs-mediated dysregulation of signal transduction pathways is associated with tumorigenesis and metastasis. Thus, a comprehensive profiling of nRTK is urgently needed to identify the members of the nRTK family that change signaling pathways, as these proteins will be key targets for drug discovery.

In conclusion, chromatin-modifying complexes and associated proteins are at the core of chromatin regulation corresponding to gene expression for organismal homeostasis. Aberrant activation or dysregulation of signal transduction pathways causes miscommunication that alters epigenetic modulators, resulting in unsubstantiated gene expression that contributes to cancer development, progression, and metastasis. In this review, we discussed several main signal transduction pathways: (1) TGF-β signaling, (2) Hippo signaling, (3) Wnt signaling, (4) Notch signaling, and (5) PI3K-AKT signaling. Those pathways are frequently dysregulated, and this dysregulation modulates chromatin-modifying complexes, leading to oncogenic genes regulation, and the promotion of tumorigenesis and metastasis. A comprehensive understanding of signaling pathways and their communication with chromatin modulators in healthy and diseased conditions is essential for the therapeutic intervention of human cancer.
